# Association between lifetime exposure to passive smoking and risk of breast cancer subtypes defined by hormone receptor status among non-smoking Caucasian women

**DOI:** 10.1371/journal.pone.0171198

**Published:** 2017-02-02

**Authors:** Loreta Strumylaite, Rima Kregzdyte, Lina Poskiene, Algirdas Bogusevicius, Darius Pranys, Roberta Norkute

**Affiliations:** 1 Neuroscience Institute, Lithuanian University of Health Sciences, Kaunas, Lithuania; 2 Department of Pathological Anatomy, Medical Academy, Lithuanian University of Health Sciences, Kaunas, Lithuania; 3 Department of Surgery, Medical Academy, Lithuanian University of Health Sciences, Kaunas, Lithuania; 4 Department of Obstetrics and Gynecology, Medical Academy, Lithuanian University of Health Sciences, Kaunas, Lithuania; Stony Brook University, Graduate Program in Public Health, UNITED STATES

## Abstract

Tobacco smoking is inconsistently associated with breast cancer. Although some studies suggest that breast cancer risk is related to passive smoking, little is known about the association with breast cancer by tumor hormone receptor status. We aimed to explore the association between lifetime passive smoking and risk of breast cancer subtypes defined by estrogen receptor and progesterone receptor status among non-smoking Caucasian women. A hospital-based case-control study was performed in 585 cases and 1170 controls aged 28–90 years. Information on lifetime passive smoking and other factors was collected via a self-administered questionnaire. Logistic regression was used for analyses restricted to the 449 cases and 930 controls who had never smoked actively. All statistical tests were two-sided. Adjusted odds ratio of breast cancer was 1.01 (95% confidence interval (CI): 0.72–1.41) in women who experienced exposure to passive smoking at work, 1.88 (95% CI: 1.38–2.55) in women who had exposure at home, and 2.80 (95% CI: 1.84–4.25) in women who were exposed at home and at work, all compared with never exposed regularly. Increased risk was associated with longer exposure: women exposed ≤ 20 years and > 20 years had 1.27 (95% CI: 0.97–1.66) and 2.64 (95% CI: 1.87–3.74) times higher risk of breast cancer compared with never exposed (P_trend_ < 0.001). The association of passive smoking with hormone receptor-positive breast cancer did not differ from that with hormone receptor-negative breast cancer (P_heterogeneity_ > 0.05). There was evidence of interaction between passive smoking intensity and menopausal status in both overall group (P = 0.02) and hormone receptor-positive breast cancer group (P < 0.05). In Caucasian women, lifetime exposure to passive smoking is associated with the risk of breast cancer independent of tumor hormone receptor status with the strongest association in postmenopausal women.

## Introduction

Tobacco smoking is defined as an agent with limited evidence in breast cancer by the International Agency for Research on Cancer [1]. This implication is related to the inconsistency of the results from numerous studies that investigated smoking and breast cancer association [2]. However, the most recent studies and meta-analyses concluded that an overall risk was plausible [3–5].

Lack of significant association in active smokers is followed by underestimation of passive smoking (mainstream smoke and sidestream smoke) as a risk factor for breast cancer. Relative to mainstream smoke, sidestream smoke contains a higher ratio of human carcinogens and some of them may induce mammary tumors [6, 7]. However, epidemiological findings on passive smoking and breast cancer association are not consistent. Some case-control [8–11] and cohort studies [12–15], particularly those that have conducted a more detailed assessment of exposure to passive smoking, reported an increase in breast cancer risk. The association of passive smoking with breast cancer was found in younger, primarily premenopausal women, whereas the risk in older or postmenopausal women was inconclusive [8, 12, 16, 17]. Other studies found little evidence in risk of breast cancer [18–21].

Experimental studies have shown that carcinogenic metals/metalloids, such as cadmium, chromium and arsenic determined in tobacco induce estrogen receptor (ER) α activation and indicate estrogen-like activity, that, in part, suggest smoking as a risk factor for breast cancer [22, 23]. This hypothesis was supported by some epidemiological studies reporting contribution of active smoking to an increased risk of ER+ breast cancer [13, 14, 24, 25]. However, the studies on passive smoking and breast cancer association in relation to hormone receptors, particularly among Caucasian women, are limited [2, 10, 26]. This study aimed to explore the association of passive smoking with risk of breast cancer subtypes defined by tumor hormone receptor status among Caucasian women.

## Materials and methods

### Study design

We performed a hospital-based case–control study of breast cancer among Caucasian women in the Hospital of Lithuanian University of Health Sciences. The cases (n = 585, presenting 86.9% of eligible cases) were women aged 28–90 years with new histologically confirmed breast cancer (C50 and D05 according to ICD10) diagnosed between 1 March 2007 and 10 January 2011, who required surgical intervention at the Department of Surgery and were free from other cancer diagnosed in the past. The controls (n = 1170, presenting 84.1% of eligible women) were women without a personal history of cancer hospitalized to other departments (Ophthalmology, Otolaryngology, Neurology, and Cardiology) of the hospital within the study period. Controls presented with a wide spectrum of non-neoplastic disorders and diseases of (a) eye (cataract, glaucoma, optic neuritis, and keratitis), (b) ear-nose-throat (otitis, sinusitis, deviation of nasal septum, tonsillitis), (c) nervous (facial and trigeminal neuritis, radiculopathy and radiculitis, epilepsy, multiple sclerosis, Parkinsonism, sleep disorders, and migraine), and (d) cardiovascular (arterial hypertension, ischemic disease, cardiomyopathy, different arrhythmias) systems. Controls were individually matched to cases by age (±5 years) in a 2:1 ratio. To assess passive smoking/breast cancer association analyses were restricted to the 449 cases and 930 controls (132, 101, 219, and 478 women from departments of Ophthalmology, Otolaryngology, Neurology, and Cardiology, respectively) who had never smoked actively. The study protocol was approved by the Kaunas Regional Biomedical Research Ethics Committee (10-01-2007 No.BE-2-1, Report No.5/2007). Written consent to complete the questionnaire and collect biological media specimens was received from each individual.

### Questionnaire and exposure assessment

Both cases and controls completed a self-administered structured questionnaire previously demonstrated to be valid and reliable for collection of demographic and socioeconomic characteristics, medical history, height and weight, family history of cancer, reproductive history, and lifestyle characteristics [27].

A woman was defined as being a passive smoker if she reported living/working with someone who smoked inside her home and/or in the workplace. Duration of exposure was assessed by the number of years of exposure at home and/or at work specified by every woman. In the case of exposure experienced both at home and at work, a longer duration reported was included in the analyses.

### Measurements of hormone receptors

The ER and progesterone receptor (PR) levels were measured in specimens of breast tumor tissue by immunohistochemistry at the Department of Pathological Anatomy [28].

### Statistical analysis

Baseline characteristics of cases and controls were summarized using means and standard deviations (SD) for continuous variables, and frequencies and percentages for categorical variables. Characteristics were compared between cases and controls using either unpaired t-test (for continuous variables) or chi-squared test (for categorical variables).

According to tumor hormone receptor status cases were stratified as follows: ER+, ER-, PR+, PR-, ER+/PR+, ER+/PR-, ER-/PR+, and ER-/PR- [29]; cases with ER-/PR+ breast cancer (n = 8) were excluded from the analyses because of insufficient statistical power.

According to the answers on the intensity of exposure to passive smoking women were grouped into 4 categories: never exposed, exposed at work only, exposed at home only, exposed at home and at work, and into 3 categories defined by duration of exposure: never exposed, ≤ 20 years, and > 20 years.

Unconditional logistic regression models were used to estimate the association between exposure to passive smoking and breast cancer subtypes calculating the odds ratios (ORs) and their 95% confidence intervals (CIs). The models were adjusted for (a) age and (b) age, number of births, estrogen-active (fertile) period, hormone therapy during menopause (never, estrogens and/or estrogens-progestin, other), family history of breast cancer in first and/or second degree of relatives (no, yes, unknown), alcohol use (never, ex-user, current), body mass index, education (specialized secondary or lower, some university or higher), marital status (single, married or living as married, separated or widowed), diabetes mellitus (absent, present), and thyroid diseases (absent, present). In these analyses, estimates of association per 1 category increase in exposure to passive smoking were obtained; for some analyses estimates for exposed at work only vs. never exposed, exposed at home only vs. never exposed, and exposed at home and at work vs. never exposed as well as for ≤ 20 years exposure vs. never exposed, and > 20 years exposure vs. never exposed were also reported.

The interaction between menopausal status and intensity or duration of exposure to passive smoking (per 1 category increase) was tested using a likelihood ratio test. Heterogeneity in the estimated associations of passive smoking with each breast cancer subtype was tested using a Cochran Q-test. The level of statistical significance was set at 0.05. All reported P-values are 2-sided. The analyses were performed using the software package Stata 10 (StataCorp LP, 2007).

## Results

Of non-smoking cases, 77.3% had invasive ductal carcinoma, 8.7% invasive lobular carcinoma, 14.0% other histological types of breast cancer. The ER+ and PR+ were determined for 66.6% and 43.2% of the cases (Table 1). The ER and PR levels were not measured in the cases with ductal and lobular carcinomas in situ (2.4%), and myoepithelial carcinoma (0.2%).

**Table 1:** Characteristics of Breast Cancer Cases and Controls among Non-Smoking Women.

Variable	Cases (n = 449)	Controls (n = 930)	P-value for difference
**Age (years) (mean, SD)**	60.34 (12.18)	59.31 (12.30)	0.14
**Education (n, %)**			
Specialized secondary or lower	287 (63.9)	658 (70.8)	
Some university or higher	162 (36.1)	272 (29.2)	0.01
**Marital status (n, %)**			
Single	20 (4.5)	45 (4.8)	
Married or living as married	265 (59.0)	566 (60.9)	
Separated or widowed	164 (36.5)	319 (34.3)	0.71
**Family history of breast cancer (n, %)**	28 (6.2)	43 (4.6)	0.20
**Age at menarche (years) (mean, SD)**	14.17 (1.73)	14.11 (1.70)	0.54
**Number of births (mean, SD)**	1.85 (1.12)	1.97 (1.13)	0.06
**Menopausal status (n, %)**			
Premenopausal	111 (24.7)	231 (24.8)	
Postmenopausal	338 (75.3)	699 (75.2)	0.96
**Estrogen-active (fertile) period (years) (mean, SD)** ^a^	34.73 (5.84)	33.66 (6.16)	<0.01
**Hormone therapy during menopause (n, %)**			
Never	281 (83.2)	622 (88.0)	
Estrogens and/or estrogens-progestin	38 (11.2)	54 (7.7)	
Other hormones (thyroxin and etc.)	19 (5.6)	23 (3.3)	0.03
**Alcohol use (n, %)**			
Never	23 (5.1)	83 (8.9)	
Ex-user	45 (10.0)	135 (14.5)	
Current	381 (84.9)	712 (76.6)	<0.01
**Passive smoking (n, %)**			
Never exposed	226 (50.3)	568 (61.1)	
Exposed at work only	63 (14.1)	158 (17.0)	
Exposed at home only	103 (22.9)	147 (15.8)	
Exposed at home and at work	57 (12.7)	57 (6.1)	<0.001
**Duration of exposure to passive smoking (years) (n, %)**			
Never exposed	226 (50.7)	568 (61.3)	
< = 20	129 (28.9)	264 (28.5)	
>20	91 (20.4)	94 (10.2)	<0.001
**Duration of exposure to passive smoking (years) (mean, SD)**^b^	20.48 (12.28)	15.73 (10.40)	<0.001
**Body mass index (kg/m**^**2**^**) (mean, SD)**	28.72 (5.73)	28.77 (5.93)	0.88
**Diabetes mellitus (n, %)**	37 (6.3)	118 (10.1)	0.01
**Thyroid diseases (n, %)**	127 (21.7)	317 (27.1)	0.01
**Tumor hormone receptors (n, %)** ^c^			
Estrogen receptor-positive	291 (66.6)		
Progesterone receptor-positive	188 (43.2)		

Abbreviations: SD, standard deviation.

^a^ Estrogen-active (fertile) period (years) = current age for non-menopausal women or age at menopause for postmenopausal women (years) minus age at menarche (years).

^b^ Duration of exposure was calculated for ever exposed to passive smoking cases and controls.

^c^ Tumor hormone receptors were determined for 437 cases. The receptors were not measured for the cases with ductal and lobular carcinomas in situ (n = 11, 2.4%), and myoepithelial carcinoma (n = 1, 0.2%).

Cases and controls were non-smoking Caucasian women of the same age, similar with respect to marital status, family history of breast cancer, age at menarche, number of births, menopausal status, and body mass index (Table 1). However, cases had higher education and a longer estrogen-active (fertile) period, more often used alcohol and hormone therapy during the menopause. Diabetes mellitus and thyroid diseases were more prevalent among controls.

Exposure to passive smoking was more often experienced by cases than controls (Table 1). Mean duration of exposure among individuals ever exposed to passive smoking was also greater in cases than in controls: 20.48 (SD = 12.28) and 15.73 (SD = 10.40) years (P *<* 0.001).

After adjustment for age and other confounders, the OR of breast cancer was 1.01 (95% CI: 0.72–1.41) in women exposed at work only, 1.88 (95% CI: 1.38–2.55) in women who experienced exposure to passive smoking at home, and 2.80 (95% CI: 1.84–4.25) in women exposed at home and at work, all compared with never exposed (Table 2). A significant increase in risk of breast cancer associated with increased duration of exposure to passive smoking was found in the adjusted model. Compared with never exposed regularly, the OR for women exposed ≤ 20 years and > 20 years was 1.27 (95% CI: 0.97–1.66) and 2.64 (95% CI: 1.87–3.74) (P_trend_ < 0.001). When stratified by menopausal status, the association appeared stronger among postmenopausal women. The interaction between menopausal status and exposure to passive smoking was significant (P = 0.02) when the intensity of exposure was analyzed (Table 2).

**Table 2:** Association between Passive Smoking and Breast Cancer among Non-Smoking Women.

Exposure to passive smoking	Cases	Controls	OR (95% CI)^a^	OR (95% CI)^b^
	n (%)	n (%)		
**All women**	449	930		
Never exposed	226 (50.3)	568 (61.1)	1	1
Exposed at work only	63 (14.1)	158 (17.0)	1.01 (0.73–1.40)	1.01 (0.72–1.41)
Exposed at home only	103 (22.9)	147 (15.8)	1.75 (1.30–2.36)	1.88 (1.38–2.55)
Exposed at home and at work	57 (12.7)	57 (6.1)	2.51 (1.69–3.74)	2.80 (1.84–4.25)
OR per 1 category increase			1.34 (1.20–1.49)	1.38 (1.23–1.55)
P_trend_			<0.001	<0.001
P_interaction_*			0.01	0.02
**Duration of exposure (years)**	446	926		
Never exposed	226 (50.7)	568 (61.3)	1	1
< = 20	129 (28.9)	264 (28.5)	1.24 (0.95–1.61)	1.27 (0.97–1.66)
>20	91 (20.4)	94 (10.2)	2.39 (1.72–3.32)	2.64 (1.87–3.74)
OR per 1 category increase			1.47 (1.26–1.72)	1.54 (1.31–1.81)
P_trend_			<0.001	<0.001
P_interaction_*			0.08	0.11
**Postmenopausal women**	338	699		
Never exposed	161 (47.6)	437 (62.5)	1	1
Exposed at work only	48 (14.2)	116 (16.6)	1.13 (0.77–1.65)	1.11 (0.75–1.63)
Exposed at home only	84 (24.9)	104 (14.9)	2.17 (1.54–3.05)	2.28 (1.60–3.25)
Exposed at home and at work	45 (13.3)	42 (6.0)	2.93 (1.85–4.63)	3.14 (1.94–5.08)
OR per 1 category increase			1.44 (1.27–1.63)	1.47 (1.29–1.67)
P_trend_			<0.001	<0.001
**Duration of exposure (years)**	336	697		
Never exposed	161 (47.9)	437 (62.7)	1	1
< = 20	97 (28.9)	178 (25.5)	1.49 (1.10–2.02)	1.49 (1.09–2.04)
>20	78 (23.2)	82 (11.8)	2.55 (1.78–3.65)	2.68 (1.83–3.92)
OR per 1 category increase			1.58 (1.32–1.87)	1.61 (1.34–1.92)
P_trend_			<0.001	<0.001
**Premenopausal women**	111	231		
Never exposed	65 (56.6)	131 (56.7)	1	1
Exposed at work only	15 (13.5)	42 (18.2)	0.72 (0.37–1.40)	0.92 (0.45–1.87)
Exposed at home only	19 (17.1)	43 (18.6)	0.91 (0.49–1.70)	1.00 (0.52–1.94)
Exposed at home and at work	12 (10.8)	15 (6.5)	1.48 (0.65–3.38)	1.89 (0.78–4.61)
OR per 1 category increase			1.04 (0.83–1.30)	1.13 (0.88–1.43)
P_trend_			0.72	0.34
**Duration of exposure (years)**	110	229		
Never exposed	65 (59.1)	131 (57.2)	1	1
< = 20	32 (29.1)	86 (37.6)	0.78 (0.47–1.29)	0.91 (0.53–1.57)
>20	13 (11.8)	12 (5.2)	1.79 (0.76–4.24)	2.57 (1.01–6.55)
OR per 1 category increase			1.07 (0.74–1.53)	1.26 (0.86–1.86)
P_trend_			0.71	0.24

Abbreviations: OR, odds ratio; CI, confidence interval.

^a^ Adjusted for age.

^b^ Further adjustment for number of births, estrogen-active (fertile) period, hormone therapy during menopause, family history of breast cancer, alcohol use, body mass index, education, marital status, diabetes mellitus, and thyroid diseases.

* P-value from likelihood ratio test of interaction between menopausal status and exposure to passive smoking (intensity or duration) (per 1 category increase).

After adjustment for age and other confounders, a significant positive association between passive smoking and breast cancer was identified independent of tumor ER and PR status (Table 3). The OR per 1 exposure (intensity and duration) category increase was 1.42 (95% CI: 1.25–1.62; P_trend_ < 0.001) and 1.54 (95% CI: 1.28–1.86; P_trend_ < 0.001), respectively, for ER+, 1.20 (95% CI: 1.00–1.43; P_trend_ = 0.05) and 1.41 (95% CI: 1.10–1.83; P_trend_ = 0.01), respectively, for ER-, 1.34 (95% CI: 1.15–1.56; P_trend_ < 0.001) and 1.47 (95% CI: 1.18–1.83; P_trend_ < 0.001), respectively, for PR+, and 1.36 (95% CI: 1.19–1.57; P_trend_ < 0.001) and 1.53 (95% CI: 1.25–1.87; P_trend_ < 0.001), respectively, for PR- breast cancer. Regardless of the exposure to passive smoking variable (intensity or duration) used in the analyses, there was no evidence of heterogeneity in the association comparing ER+ and ER- (P > 0.05) or PR+ and PR- breast cancer (P > 0.05). The association appeared stronger among postmenopausal women than premenopausal women. Significant interaction between menopausal status and intensity of exposure to passive smoking was found for both ER+ (P = 0.03) and PR+ (P = 0.02) breast cancer (Table 3).

**Table 3:** Association between Passive Smoking and Breast Cancer by Estrogen Receptor and Progesterone Receptor Status among Non-Smoking Women.

Exposure to passive smoking	n controls	ER+		ER-		P_heter_*	PR+		PR-		P_heter_*
		n cases	OR (95% CI) ^a^	n cases	OR (95% CI) ^a^		n cases	OR (95% CI) ^a^	n cases	OR (95% CI) ^a^	
**All women**	930	291		146			188		249		
Never exposed	568	140	1	82	1		97	1	125	1	
Exposed at work only	158	38	0.95 (0.63–1.43)	23	1.05 (0.63–1.74)		22	0.81 (0.49–1.34)	39	1.11 (0.74–1.68)	
Exposed at home only	147	73	2.01 (1.42–2.86)	28	1.45 (0.89–2.35)		45	1.77 (1.18–2.68)	56	1.88 (1.29–2.75)	
Exposed at home and work	57	40	3.02 (1.89–4.84)	13	1.70 (0.86–3.35)		24	2.63 (1.52–4.58)	29	2.55 (1.53–4.26)	
OR per 1 category increase			1.42 (1.25–1.62)		1.20 (1.00–1.43)			1.34 (1.15–1.56)		1.36 (1.19–1.57)	
P_trend_			<0.001		0.05	0.13		<0.001		<0.001	0.86
P_interaction_**			0.03		0.32			0.02		0.21	
**Duration of exposure (years)**	926	289		145			188		246		
Never exposed	568	140	1	82	1		97	1	125	1	
< = 20	264	88	1.35 (0.99–1.85)	37	0.97 (0.63–1.49)		51	1.13 (0.77–1.65)	74	1.30 (0.93–1.82)	
>20	94	61	2.59 (1.74–3.86)	26	2.49 (1.47–4.22)		40	2.54 (1.60–4.02)	47	2.56 (1.67–3.92)	
OR per 1 category increase			1.54 (1.28–1.86)		1.41 (1.10–1.83)			1.47 (1.18–1.83)		1.53 (1.25–1.87)	
P_trend_			<0.001		0.01	0.61		<0.001		<0.001	0.80
P_interaction_**			0.09		0.71			0.06		0.61	
**Postmenopausal women**	699	229		101			135		195		
Never exposed	437	103	1	56	1		64	1	95	1	
Exposed at work only	116	30	1.04 (0.65–1.66)	16	1.10 (0.60–2.02)		14	0.81 (0.43–1.50)	32	1.23 (0.78–1.95)	
Exposed at home only	104	64	2.53 (1.70–3.76)	19	1.59 (0.87–2.87)		39	2.47 (1.54–3.96)	44	2.07 (1.34–3.20)	
Exposed at home and work	42	32	3.30 (1.93–5.65)	10	2.03 (0.93–4.44)		18	2.99 (1.56–5.72)	24	2.82 (1.59–5.04)	
OR per 1 category increase			1.52 (1.31–1.76)		1.26 (1.02–1.56)			1.48 (1.24–1.77)		1.42 (1.21–1.66)	
P_trend_			<0.001		0.03	0.16		<0.001		<0.001	0.73
**Duration of exposure (years)**	697	228		100			135		193		
Never exposed	437	103	1	56	1		64	1	95	1	
< = 20	178	71	1.63 (1.14–2.34)	24	1.06 (0.62–1.78)		36	1.38 (0.87–2.18)	59	1.50 (1.02–2.19)	
>20	82	54	2.67 (1.73–4.11)	20	2.35 (1.29–4.31)		35	2.72 (1.64–4.52)	39	2.43 (1.51–3.89)	
OR per 1 category increase			1.62 (1.32–2.00)		1.44 (1.07–1.93)			1.59 (1.25–2.04)		1.54 (1.23–1.92)	
P_trend_			<0.001		0.02	0.50		<0.001		<0.001	0.84
**Premenopausal women**	231	62		45			53		54		
Never exposed	131	37	1	26	1		33	1	30	1	
Exposed at work only	42	8	0.81 (0.33–1.99)	7	1.06 (0.40–2.78)		8	0.90 (0.36–2.22)	7	0.93 (0.36–2.41)	
Exposed at home only	43	9	0.75 (0.32–1.78)	9	1.12 (0.45–2.75)		6	0.59 (0.22–1.58)	12	1.44 (0.64–3.23)	
Exposed at home and work	15	8	2.08 (0.74–5.89)	3	1.33 (0.30–5.86)		6	2.02 (0.66–6.18)	5	1.92 (0.56–6.59)	
OR per 1 category increase			1.08 (0.80–1.46)		1.09 (0.77–1.55)			1.00 (0.73–1.39)		1.22 (0.89–1.67)	
P_trend_			0.61		0.63	0.98		0.98		0.22	0.41
**Duration of exposure (years)**	229	61		45			53		53		
Never exposed	131	37	1	26	1		33	1	30	1	
< = 20	86	17	0.77 (0.39–1.54)	13	0.94 (0.44–2.02)		15	0.81 (0.40–1.66)	15	0.98 (0.47–2.04)	
>20	12	7	2.09 (0.69–6.36)	6	2.95 (0.84–10.40)		5	1.92 (0.56–6.59)	8	3.37 (1.09–10.45)	
OR per 1 category increase			1.10 (0.68–1.78)		1.37 (0.80–2.34)			1.02 (0.61–1.73)		1.46 (0.89–2.39)	
P_trend_			0.71		0.25	0.55		0.93		0.13	0.33

Abbreviations: OR, odds ratio; CI, confidence interval; ER+, estrogen receptor-positive; ER-, estrogen receptor-negative; PR+, progesterone receptor-positive; PR-, progesterone receptor-negative.

^a^ Adjusted for age, number of births, estrogen-active (fertile) period, hormone therapy during menopause, family history of breast cancer, alcohol use, body mass index, education, marital status, diabetes mellitus, and thyroid diseases.

* P-value from Cochran Q test of heterogeneity in the associations between exposure to passive smoking (intensity or duration) (per 1 category increase) and (1) ER+ or ER- breast cancer, (2) PR+ or PR- breast cancer.

** P-value from likelihood ratio test of interaction between menopausal status and exposure to passive smoking (intensity or duration) (per 1 category increase).

There were significant positive associations between passive smoking and (1) ER+/PR-, (2) ER+/PR+, and (3) ER-/PR- breast cancer (Table 4). A 1 category increase in passive smoking intensity was associated with an OR of 1.56 (95% CI: 1.28–1.89; P_trend_ < 0.001) for ER+/PR- breast cancer, which was different from the passive smoking/ER-/PR- association (P_heterogeneity_ = 0.05), with an OR of 1.34 (95% CI: 1.15–1.57; P_trend_ < 0.001) for ER+/PR+ breast cancer, not significantly different from the passive smoking/ER-/PR- association (p_heterogeneity_ = 0.35), and with an OR of 1.20 (95% CI: 1.00–1.44; P_trend_ = 0.05) for ER-/PR- breast cancer. Increased duration of exposure was related to higher risk of breast cancer independently of tumor hormone receptor status (P_heterogeneity_ > 0.05). When stratified by menopausal status, the association was stronger among postmenopausal women than premenopausal women; however, significant interaction between menopausal status and intensity of exposure to passive smoking was found only for ER+PR+ breast cancer (P = 0.02) (Table 4).

**Table 4:** Association between Passive Smoking and Breast Cancer Defined by Joint Hormone Receptor Status among Non-Smoking Women.

Exposure to passive smoking	n controls	ER+/PR-		ER+/PR+		ER-/PR-	
		n cases	OR (95% CI) ^a^	n cases	OR (95% CI) ^a^	n cases	OR (95% CI) ^a^
**All women**	930	111		180		138	
Never exposed	568	48	1	92	1	77	1
Exposed at work only	158	16	1.10 (0.60–2.03)	22	0.85 (0.51–1.41)	23	1.11 (0.67–1.84)
Exposed at home only	147	31	2.56 (1.54–4.28)	42	1.73 (1.13–2.64)	25	1.37 (0.83–2.26)
Exposed at home and at work	57	16	3.64 (1.86–7.12)	24	2.72 (1.56–4.74)	13	1.77 (0.90–3.49)
OR per 1 category increase			1.56 (1.28–1.89)		1.34 (1.15–1.57)		1.20 (1.00–1.44)
P_trend_			<0.001		<0.001		0.05
P_heterogeneity_*			0.05		0.35		
P_interaction_**			0.82		0.02		0.31
**Duration of exposure (years**)	926	109		180		137	
Never exposed	568	48	1	92	1	77	1
< = 20	264	39	1.73 (1.09–2.75)	49	1.15 (0.78–1.69)	35	0.98 (0.63–1.52)
>20	94	22	2.64 (1.45–4.79)	39	2.53 (1.59–4.02)	25	2.46(1.44–4.21)
OR per 1 category increase			1.63 (1.23–2.16)		1.48 (1.18–1.84)		1.42 (1.10–1.84)
P_trend_			0.001		0.001		<0.01
P_heterogeneity_*			0.48		0.84		
P_interaction_**			0.73		0.07		0.83
**Postmenopausal women**	699	97		132		98	
Never exposed	437	41	1	62	1	54	1
Exposed at work only	116	16	1.33 (0.71–2.50)	14	0.83 (0.45–1.56)	16	1.14 (0.62–2.09)
Exposed at home only	104	26	2.56 (1.46–4.50)	38	2.47 (1.53–3.99)	18	1.55 (0.85–2.83)
Exposed at home and at work	42	14	3.59 (1.74–7.41)	18	3.04 (1.59–5.83)	10	2.08 (0.95–4.54)
OR per 1 category increase			1.55 (1.26–1.91)		1.49 (1.24–1.78)		1.26 (1.02–1.57)
P_trend_			<0.001		<0.001		0.03
P_heterogeneity_*			0.18		0.26		
**Duration of exposure (years)**	697	96		132		97	
Never exposed	437	41	1	62	1	54	1
< = 20	178	35	1.91 (1.16–3.14)	36	1.43 (0.90–2.26)	24	1.09 (0.64–1.85)
>20	82	20	2.54 (1.35–4.75)	34	2.68 (1.60–4.47)	19	2.28 (1.23–4.22)
OR per 1 category increase			1.63 (1.21–2.18)		1.59 (1.24–2.04)		1.43 (1.06–1.93)
P_trend_			0.001		<0.001		0.02
P_heterogeneity_*			0.55		0.59		
**Premenopausal women**	231	14		48		40	
Never exposed	131	7	1	30	1	23	1
Exposed at work only	42	0	-	8	0.94 (0.38–2.33)	7	1.17 (0.44–3.11)
Exposed at home only	43	5	2.12 (0.56–8.06)	4	0.39 (0.13–1.23)	7	0.98 (0.37–2.60)
Exposed at home and at work	15	2	3.12 (0.42–22.88)	6	1.97 (0.64–6.05)	3	1.45 (0.32–6.46)
OR per 1 category increase			1.54 (0.88–2.68)		0.98 (0.70–1.38)		1.11 (0.77–1.60)
P_trend_			0.13		0.91		0.59
P_heterogeneity_*			0.29		0.71		
**Duration of exposure (years)**	229	13		48		40	
Never exposed	131	7	1	30	1	23	1
< = 20	86	4	0.97 (0.23–4.11)	13	0.72 (0.34–1.51)	11	0.89 (0.40–2.00)
>20	12	2	2.09 (0.28–15.47)	5	1.78 (0.52–6.08)	6	3.26 (0.92–11.46)
OR per 1 category increase			1.40 (0.57–3.44)		1.03 (0.60–1.76)		1.40 (0.81–2.43)
P_trend_			0.46		0.92		0.23
P_heterogeneity_*			0.99		0.42		

Abbreviations: OR, odds ratio; CI, confidence interval; ER+/PR-, estrogen receptor-positive and progesterone receptor-negative; ER+/PR+, estrogen receptor-positive and progesterone receptor-positive; ER-/PR-, estrogen receptor-negative and progesterone receptor-negative.

^a^ Adjusted for age, number of births, estrogen-active (fertile) period, hormone therapy during menopause, family history of breast cancer, alcohol use, body mass index, education, marital status, diabetes mellitus, and thyroid diseases.

* P-value from Cochran Q test of heterogeneity in the associations between exposure to passive smoking (intensity or duration) (per 1 category increase) and either ER-PR- or one of the following ER+PR-, ER+PR+ breast cancer.

** P-value from likelihood ratio test of interaction between menopausal status and exposure to passive smoking (intensity or duration) (per 1 category increase).

## Discussion

In this hospital-based case-control study of breast cancer among non-smoking Caucasian women, we observed a 1.9-fold increase in breast cancer risk for women exposed to passive smoking at home, a 2.8-fold increase for those exposed at home and at work, and a 2.6-fold increase in breast cancer risk for women experienced passive smoking > 20 years. Two large prospective studies completed in Europe and the US observed greater risk of developing breast cancer in women exposed to passive smoking [14, 25]. Increased risk has been seen in some other studies [8–13, 15, 26, 30], but not in all [18–21, 31, 32]. Higher risk of breast cancer related to increasing duration of exposure to passive smoking was observed in a Canadian case-control study [16] and in two most recently published studies [10, 26]. However, other studies failed to find the association [8, 9, 31]. Some meta-analyses included the latest surveys have shown significant passive smoking/breast cancer association with risk varying from 1.2 to 2.2 [5, 33–35], but those included only cohort studies [19, 36] have found little evidence on passive smoking and breast cancer association. Significant risk of breast cancer was not identified in the previous meta-analyses of both cohort studies and case-control studies with missing information on important passive exposure sources [37].

As breast cancer has different subtypes according to hormone receptor status, their etiological and risk factors may vary [2]. Our results showed that passive smoking/breast cancer association was independent of tumor hormone receptor, although suggestive heterogeneity in the association comparing ER+/PR- and ER-/PR- breast cancer (P = 0.05) was found. Just as we, Li et al [26] also observed increased risk of breast cancer independent of tumor hormone receptor status. Other two studies (cohort and case-control) showed an elevated risk of ER+/PR+ breast for women who had been exposed to passive smoking, however, heterogeneity of the association was not reported [10, 14]. No significant association between passive smoking and breast cancer hormone receptor status was found by other authors [21, 25].

Until now epidemiological studies provided little evidence for passive smoking and breast cancer association in postmenopausal women [12, 13, 19, 33]. However, some authors, just as we, identified greater risk of breast cancer among postmenopausal women [26]. A positive association of passive smoking with postmenopausal breast cancer was found in two cohort studies [25, 30]. Luo et al [25] reported a 32% excess risk of breast cancer in postmenopausal women who had never smoked but had the most extensive exposure to passive smoking (≥ 30 years’ exposure at home and ≥ 10 years’ exposure at work) compared with those who had never been exposed to passive smoking (adjusted hazard ratio = 1.32, 95% CI: 1.04, 1.67) [25]. An elevated risk at the highest level of exposure for women exposed in the adulthood, mainly among postmenopausal women (hazard ratio = 1.25, 95% CI: 1.01, 1.56), was found in the California Teachers Study [30]. But the Million Women Study did not show any association [19]. Some studies found passive smoking to be associated with increased risk of premenopausal breast cancer, but not in postmenopausal [8, 12, 13]; other studies found limited evidence on the association in both premenopausal and postmenopausal women [15, 38, 39]. We also observed potential interaction of passive smoking with menopausal status in both overall group and hormone receptor-positive breast cancer.

Inconsistency of reported findings could be related to some issues such as the relatively small number of cases in subgroup analyses [12, 13] or limited and underestimated information on lifetime exposure to passive smoking [8, 12, 13, 15, 19]. As a consequence, if passive smoking increases the risk of breast cancer, inclusion of women experienced exposure in the reference category might have diluted association. Some cohort studies made exposure assessment and collected information on menopausal status only at baseline [12, 15, 25]. Therefore, possible changes of exposure and menopausal status during the follow-up period could give misclassification of individuals by these factors.

An increased risk of postmenopausal cancer determined in the studies, including the present one, opposes anti-estrogenic effects of tobacco smoke [2]. Although passive smoking can be related to decreased levels of estrogens [40], some constituents of tobacco smoke (metals, metalloids and etc.) that tend to accumulate in the human body have estrogen-like activity [23]. This implication is based on experimental studies demonstrating the ability of some metals and metalloids to mimic estradiol and activate the ER. Therefore, environmental exposure to metallo-estrogens may increase the risk of breast cancer [41–43]. Moreover, higher levels of some metals such as cadmium, chromium, and nickel have been reported in breast tumor of cancer patients [44–46]. ER+ cases also demonstrated higher content of cadmium in breast tumor compared to ER- cases [46]. Epidemiological studies showed an increased risk of breast cancer in relation to increasing urinary cadmium [47–50] that is long-term exposure biomarker [51]. In addition, many of the metals can initiate carcinogenesis inducing oxidative stress [23, 52].

Epidemiological findings on breast cancer risk related to passive smoking at work are not consistent. We, as well as other authors [12, 14, 26, 30], found little evidence of association between passive smoking at work and breast cancer. Policies banning smoking in the workplaces and, consequently, decreased number of women exposed to passive smoking at work can be one of the explanations. Positive passive smoking/breast cancer association was found in two Chinese studies [39, 53]. However, the authors of Shanghai Breast Cancer Study could not disprove other exposures in the workplace possibly related to greater risk of breast cancer [39].

We assessed lifetime exposure to passive smoking at home, without distinction the exposure experienced in childhood. The studies published previously showed inconclusive results on passive smoking during childhood and breast cancer association where some authors [53, 54] but not all [14, 19, 25, 32, 38] found the association. Inconsistent results could be related to exposure assessment. To obtain detailed and correct information on passive smoking exposure, particularly during childhood, using indirect measures (questionnaires) is very difficult, because most of the individuals do not calculate and/or can not remember how many cigarettes were smoked by their parents or family members at home, how many hours they experienced such exposure every day and etc. Furthermore, this information could vary over time. Consequently, not accurate information obtained can lead to false classification of the individuals due to exposure.

In this study, in which controls were individually matched to cases by age, to estimate the association we used unconditional logistic regression (adjusted for age and other risk factors) rather than conditional logistic regression. Conditional logistic regression was more powerful and showed stronger effects in the overall sample; however, when considering different breast cancer subtypes defined by hormone receptors and, especially, by menopausal status, the small number of individuals with certain breast cancer subtypes led to wide CIs around the estimated associations, especially for premenopausal women.

One of the main limitations of this case-control is using hospital’s patients free of cancer as controls, but not randomly recruited controls, i.e. representatives of the general population in terms of health. This recruitment of controls is prone to selection bias because the controls are different from the population they are meant to represent. However, in this study, cases and controls were elderly (age mean 60.34 ± 12.18 of the cases and 59.31 ± 12.30 of the controls) and postmenopausal women (75% of cases and controls) that begin to have health related issues represented by the general population. Therefore recruitment of controls from different departments of the hospital may reflect health issues in this population. Some authors reported that hospital controls were comparable with population controls for most demographic and lifestyle characteristics [55].

Another limitation of case-control studies is that information on exposure is subject to recall bias that deals with the systematic difference between cases and controls in the accuracy of the information reported, because cases are more likely to recall their exposure to potential risk factors. To avoid recall bias in the assessment of lifetime exposure to passive smoking, firstly, we did not disclose study hypothesis. Secondly, in hospital-based case-control all women were with “a disease” and, therefore, had the same attitude to report information on potential risk factors. Thirdly, we did not ask women for very detailed information (a number of cigarettes smoked by parents or other family members, passive smoking exposure in hours, and etc.) that is difficult to recollect.

We also had relatively small numbers of individuals, mainly cases, in subgroups by menopausal status and tumor hormone receptor status that limited our analyses.

However, an increase in risk of breast cancer related to passive smoking that we observed in this study could not be explained by biases and confounding factors common in case-control studies or by chance because, firstly, we invited all the patients that met selection criteria within the study period at the departments of the hospital selected randomly and did not disclose the hypothesis. Both cases and controls were representative of patients in a hospital which provides medical services for the population, and had equal conditions and presumptions answering to the same questions. Therefore, the probability of systematic differences in reporting between cases and controls was low. Secondly, we collected data on all aspects of lifetime exposure to passive smoking (anytime residential in childhood or later and occupational) and its duration which enabled us to establish a reference group that had been unexposed to either active or passive smoking. Thirdly, we adjusted for all known confounders for breast cancer.

In conclusion, the study among Caucasian women provides evidence on the association between lifetime exposure to passive smoking and risk of breast cancer independent of tumor hormone receptor status with a stronger risk demonstrated among postmenopausal women. Further studies are necessary to define whether the association with passive smoking varies by menopausal status independently of other risk factors. Since passive smoking is a modifiable factor, the findings are very important to public health and primary health care workers that could inform and advise women to avoid this exposure.

## Supporting information

S1 Data Data BC Passive Smoking. (DTA)
